# Free latissimus dorsi flap for upper extremity reconstruction in a 9-month-old

**DOI:** 10.1080/23320885.2021.1947141

**Published:** 2021-07-02

**Authors:** Ryan D. Wagner, Jacqueline S. Yang, Brittany E. Bryant, William C. Pederson, Shayan A. Izaddoost

**Affiliations:** a Division of Plastic Surgery, Department of Surgery, Baylor College of Medicine, Houston, TX, USA; b Department of Plastic Surgery, Kelsey-Seybold Clinic, Houston, TX, USA

**Keywords:** Free flap, free tissue transfer, latissimus dorsi, general anesthesia, pediatric surgery

## Abstract

Successful outcomes for free tissue transfer are well-documented in pediatric patients but less so in infants. Challenges with infants are unique and include implications of prolonged anesthetic exposure. We present a 9-month-old female who underwent a free latissimus dorsi flap to reconstruct a congenital upper extremity lesion threatening limb development.

## Introduction

Free tissue transfer can be one of the most powerful reconstructive procedures and occupies the top rung on the reconstructive ladder. Free tissue transfer in the pediatric population is now routinely performed at many institutions for a wide range of indications. With advances in technology and microsurgical technique, success rates are comparable to the adult population [1–3]. However, there is far less literature on free tissue transfer in infants along with a variety of complicating factors. In addition to greater technical difficulty, lower functional reserve, and a challenging postoperative recovery process, there is the added consideration of operative time and long-term neurological consequences of exposure to general anesthetics in infants. Free flaps can be one of the lengthiest reconstructive surgeries with the potential for additional surgeries for emergent takeback during the initial postoperative period [4].

In 2016 and 2017, the FDA released drug safety communications warning that surgeries in children younger than 3 years involving multiple or lengthy exposures to general anesthesia and sedative drugs could affect brain development. Furthermore, these warnings suggested sedation over 3 hours could be linked to long-term behavioral or learning deficits [5,6]. These conclusions were based primarily on animal studies that found widespread neuronal death after hours of prolonged NMDA inhibitor anesthesia usage [7,8]. Subsequent studies have shown that GABA agonists can also negatively impact neurodevelopment [9]. While this data in mice, rats, and non-human primates presents significant concerns for the field of pediatric anesthesia and surgery, the implications of these neurotoxic effects have not yet been clearly translated into human metrics [10,11]. With this new data, reconstructive surgeons have to be extremely diligent when planning large reconstructive procedures in the young pediatric population and always weigh the risks and benefits of early intervention.

Here we present a case of a 9-month-old female with an extensive congenital soft-tissue defect of the right forearm who underwent a latissimus dorsi free myocutaneous flap to provide durable soft tissue coverage, prevent growth restriction, and avoid further functional loss.

## Case presentation

**Figure 1.**
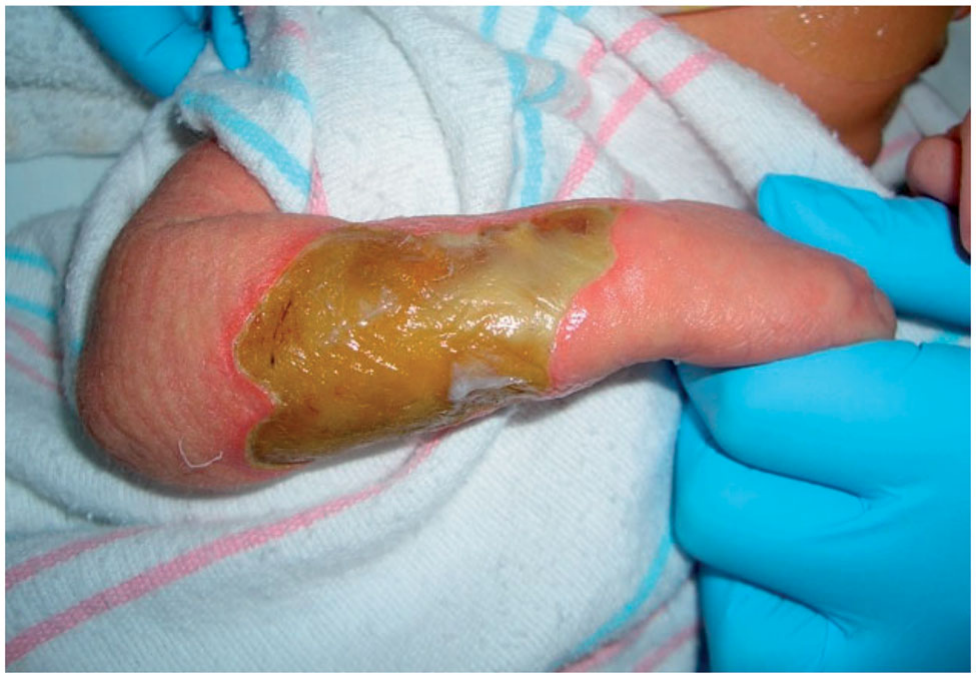
The initial full thickness dorsoulnar lesion of the right forearm.

A female infant was born at 39 weeks to a 32-year-old mother via in-utero fertilization, with pregnancy complicated by diet-controlled gestational diabetes and oligohydramnios. The patient was noted to have a large full-thickness right dorsal hand and dorsoulnar forearm lesion measuring 4.5 cm × 6.5 cm (Figure 1). Initially, movement in the extremity was unaffected except for mild weakness of extension noted in the thumb and index fingers. A biopsy was unremarkable, and there were no significant radiographic or ultrasound findings. A chromosomal microarray revealed a small duplication on Xq13.2 which was of indeterminate significance after consultation with the Genetics Team. After a multidisciplinary diagnostic approach, neonatal compartment syndrome, amniotic band syndrome, or aplasia cutis were suspected etiologies [12].

The wound was allowed to heal by secondary intention with local wound care; however, a severe scar contracture developed. At two months of age, scar release and two z-plasties were performed under general anesthesia to lengthen the scar and allow for growth of the forearm.

**Figure 2.**
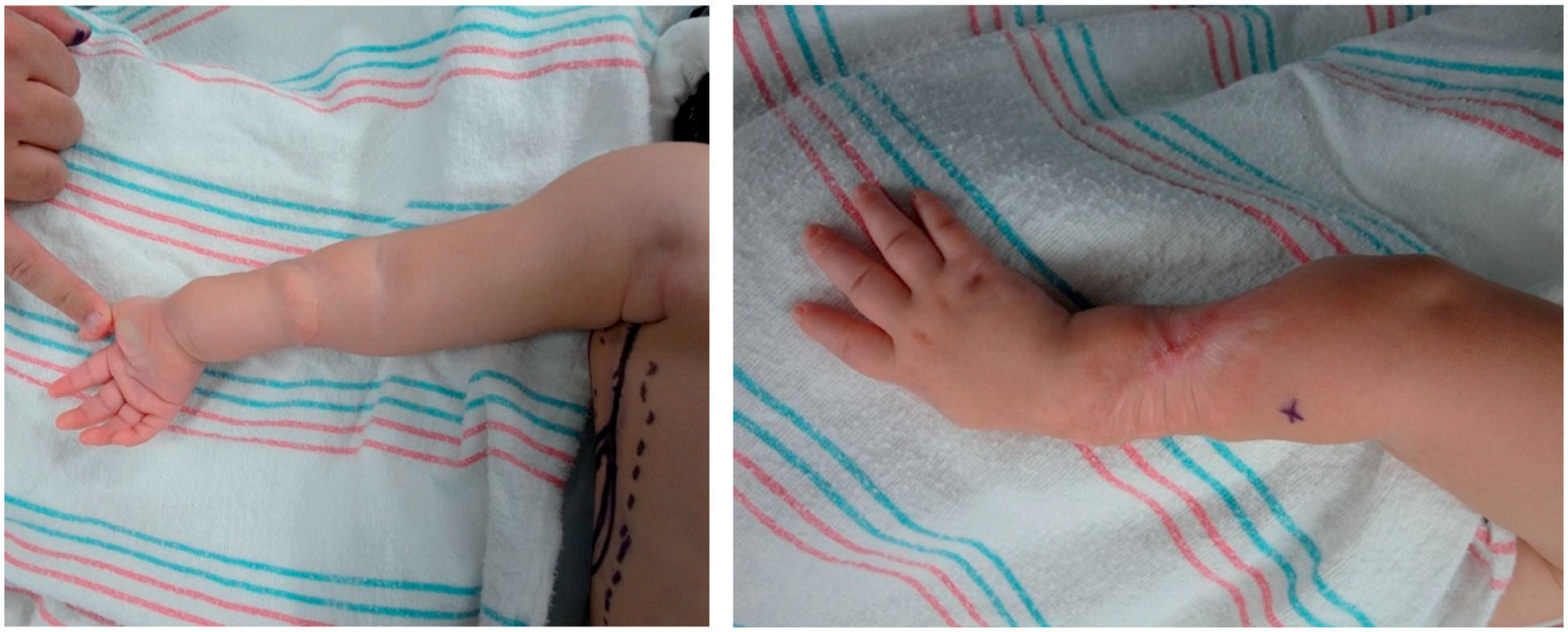
Preoperative photographs of the right forearm after scar release and two z-plasties with persistent severe contracture.

**Figure 3.**
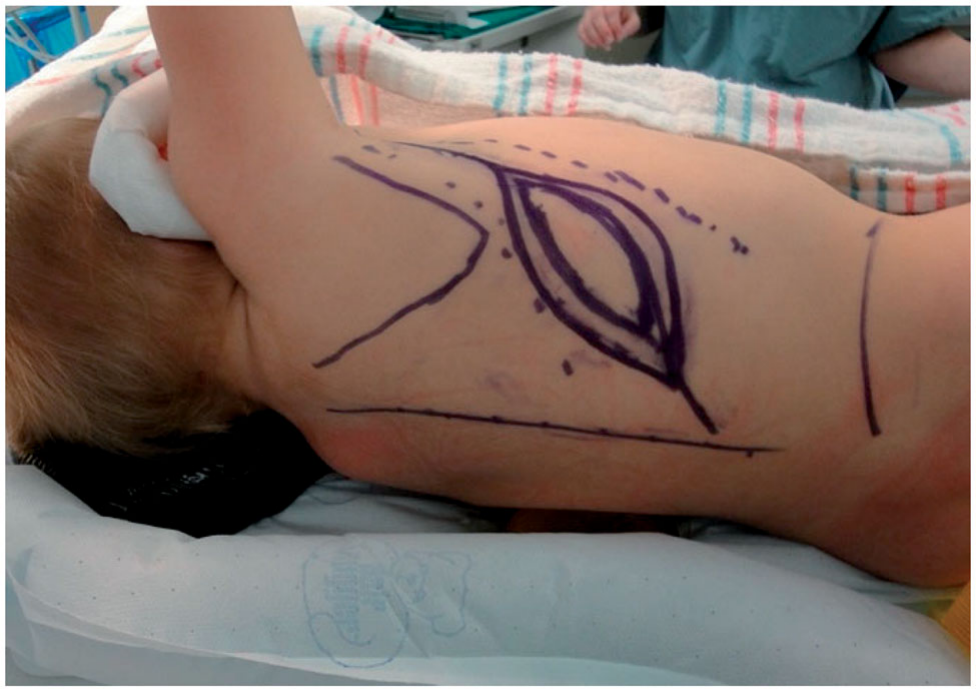
Preoperative markings for a left latissimus dorsi flap with skin paddle.

**Figure 4.**
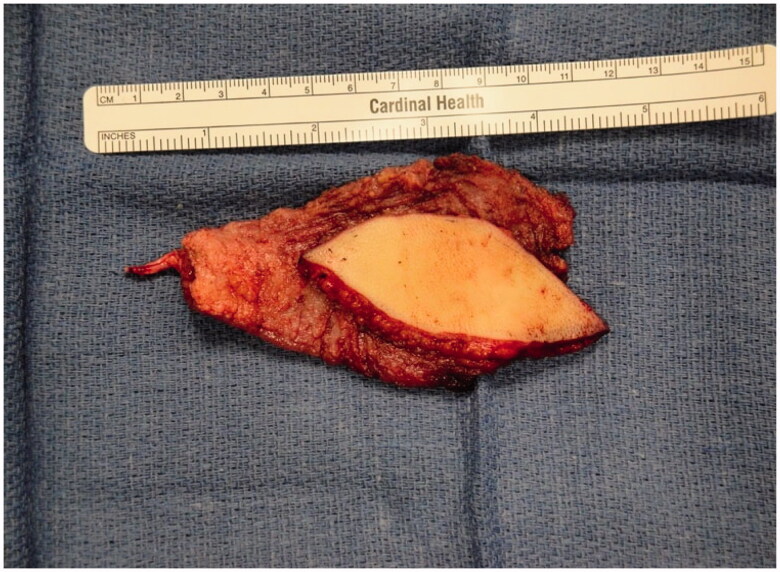
Intra-operative photograph of the latissimus dorsi flap after dissection and before anastomosis and inset.

**Figure 5.**
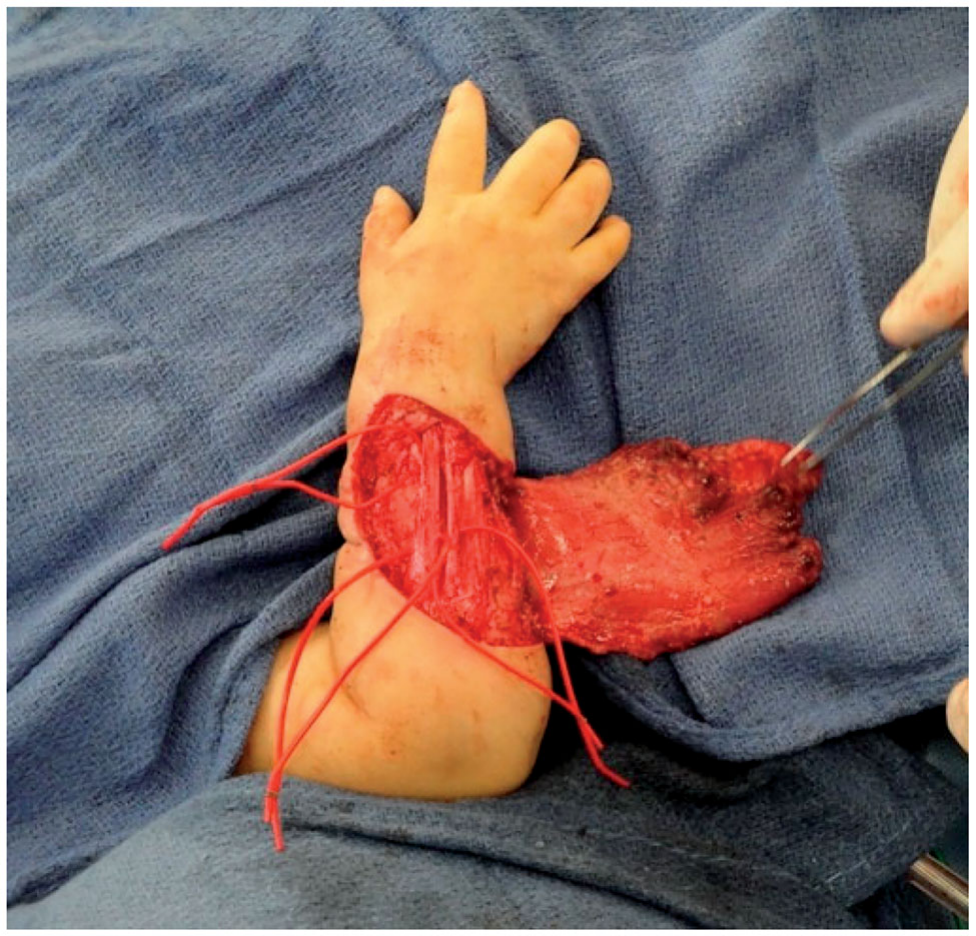
Intra-operative photograph after extensor tenolysis and prior to final flap inset.

**Figure 6.**
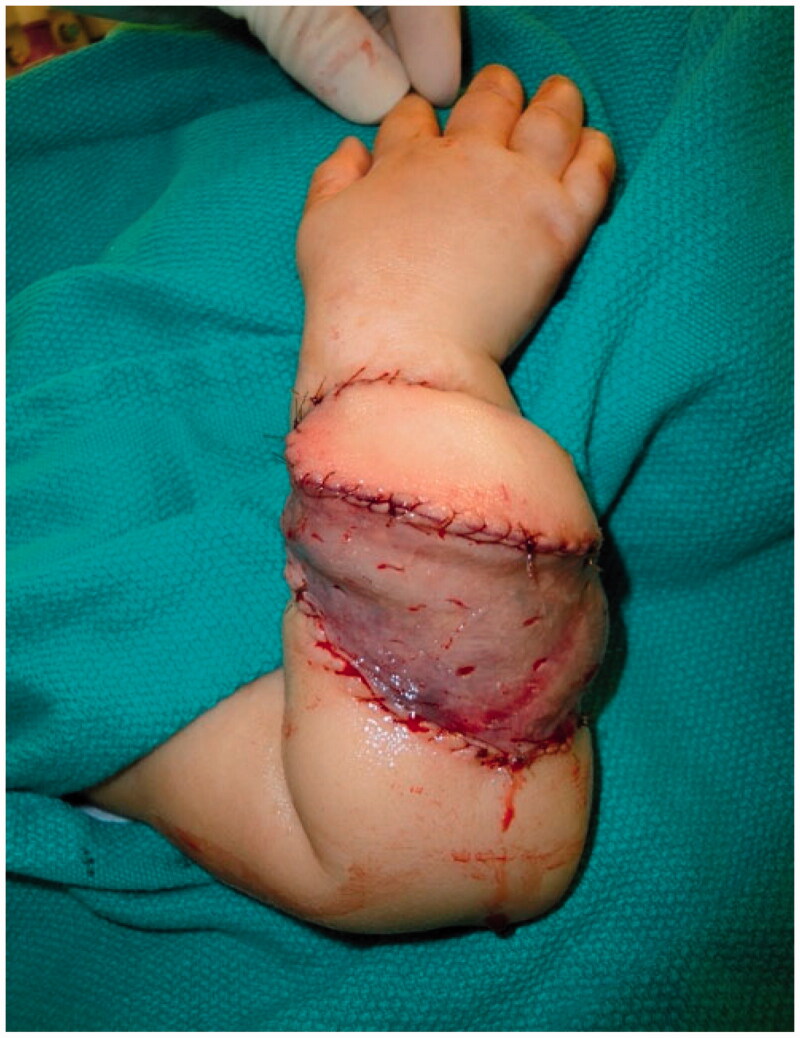
Intra-operative photograph of the final inset of the latissimus dorsi flap with a skin paddle and split thickness skin graft.

**Figure 7.**
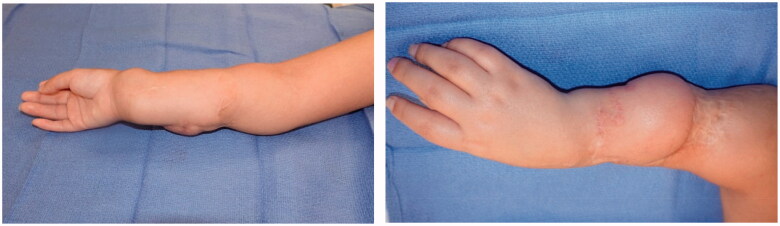
Long-term follow-up at age nine when the patient presented for flap revision with z-plasties and debulking.

Improvement was temporary and by 9 months of age, the patient developed ulnar deviation from a lack of growth at the ulna and restriction to wrist and finger extension likely secondary to scarring and adhesions (Figure 2). After a thorough discussion with the parents, the patient underwent complete scar excision, extensive tenolysis to the entire extensor compartment, and coverage with a latissimus dorsi free flap with a 9 × 5 cm split-thickness skin graft (Figures 3–6). Total operative time was just over 9 hours. The patient remained intubated in the NICU for 5 days postoperatively to allow for flap monitoring and initial healing. A continuous heparin infusion of 5 units/kg/h was administered for 10 days while in the hospital. The patient underwent a routine recovery from the operation without complication. The patient required several subsequent surgeries including distraction lengthening of the ulna at age 4, followed by a palmaris longus to extensor pollicis longus tendon transfer and z-plasty scar revision at age 6, and finally a flap debulking procedure at age 9 (Figure 7).

## Discussion

The safety, success rates, and outcomes of free tissue transfer in the pediatric patient population have been well documented in the literature by numerous reviews and case series [1,2,13]. Free flaps are utilized to reconstruct a wide range of pediatric defects – including oncologic, traumatic, and congenital. However, significantly less literature is available on the implementation of free tissue transfer in reconstruction for infants. Indications and outcomes are largely limited to a select few case reports [14–17]. The decision to proceed with free tissue transfer, in this case, was determined by the extensive scarring and impaired skeletal growth. Skin grafting or the use of dermal regeneration templates would not bring in new well-vascularized tissue with a concern for subsequent contracture with later growth. The size of the lesion and the location on the dorsal forearm with underlying extensor tendons prohibited local options for coverage. Without this operation, the patient would have likely developed a permanent and significant functional deformity. The latissimus dorsi flap was chosen as the donor because of the size of the defect, low donor site morbidity, relative ease of dissection, and consistent vascular anatomy with a large caliber vessel in the thoracodorsal artery. The size and availability of donor tissues are problematic in the infant population and fasciocutaneous flaps can be too small for coverage of large wounds. The latissimus dorsi flap was the donor site of choice for many of the published reports of free tissue transfer in infants [14,16,17].

Microsurgery in the young pediatric patient population can be technically demanding with smaller vessel diameters and unique postoperative challenges. In a series of free perforator flaps in children, Landuyt et al. reported a successful TDAP flap performed for coverage after a transmetatarsal amputation for circumferential foot necrosis after iatrogenic injury in a 10-week premature infant [18]. While no minimum age has been reported as a cutoff to performing free tissue transfer, there are several considerations that may be unique to infants. The general pediatric patient requiring free tissue transfer often has fewer comorbidities, a greater functional reserve, and lacks vascular disease compared to their adult counterparts, however, this does not necessarily hold true for infants [1,13]. Indications for free tissue transfer in infants are often congenital in nature and the patients can have associated defects or anomalies complicating peri-operative or post-operative care. Further, infants do not possess the functional reserve present in healthy older pediatric patients. Intra-operatively the issue of vasospasm has been debated, however, less is known about this in infants [1,2,19]. Post-operative care can be particularly challenging and requires diligent communication and oversight by parents and nursing staff as well as a well-coordinated multidisciplinary approach to ICU care.

Additionally, in infants and children under 3 years, the effects of prolonged anesthesia need to be carefully considered. Every effort should be made to decrease operative time, the time between induction and the start of surgery, and time between the end of surgery and the end of anesthesia [7]. If space and positioning allow, a two-team approach should also be considered. Many anesthetic agents have been shown to significantly alter brain development in preclinical trials primarily based in animal models [10]. Those most at risk are young patients undergoing long procedures or multiple surgeries, although longitudinal data in human subjects is lacking [7,10,11]. The Mayo Anesthesia Safety in Kids study, the first matched cohort longitudinal study to address this issue, found no significant relationship between generalized intelligence and multiple anesthetic events in young children however there was an association with processing speed and fine motor coordination [20]. Until further high-quality research is conducted, plastic surgeons operating on children younger than three for large reconstructive problems need to carefully determine if reconstruction can be delayed or if alternative, shorter, treatments are available which can achieve the same reconstructive goal.

## Conclusion

We presented one of the few cases of a free latissimus flap for upper extremity reconstruction in an infant with a discussion on the challenges of free tissue transfer in the young pediatric population. For patients, parents, and physicians, it is important to understand the risks involved in these extensive reconstructive procedures. Surgeons should use their best judgment and weigh the benefits of the reconstructive outcome with the potential risks of anesthesia for infants potentially susceptible to neurotoxicity.
